# Kusankha Pamodzi: Health Care Decision-Making Preferences Among Patients with Cancer in Malawi

**DOI:** 10.1089/pmr.2023.0002

**Published:** 2023-04-27

**Authors:** Alyssa E. Tilly, April Evans, Jane S. Chen, Agness Manda, Ande Salima, Samuel Bingo, Maria Chikasema, Katherine D. Westmoreland

**Affiliations:** ^1^ UNC Project-Malawi, Lilongwe, Malawi.; ^2^ Department of Medicine, University of North Carolina at Chapel Hill, Chapel Hill, North Carolina, USA.; ^3^ Department of Pediatrics, University of North Carolina at Chapel Hill, Chapel Hill, North Carolina, USA.; ^4^ Institute for Global Health and Infectious Diseases, University of North Carolina at Chapel Hill, Chapel Hill, North Carolina, USA.; ^5^ Malawi Ministry of Health, Lilongwe, Malawi.

**Keywords:** cancer, communication, decision making, goals of care, Malawi

## Abstract

**Background:**

Oncology teams are encouraged to include patient preferences and goals of care in determining appropriate treatment courses. There are no existing data from Malawi exploring decision-making preferences among cancer patients.

**Methods:**

In the oncology clinic in Lilongwe, Malawi, 50 patients were surveyed for decision making.

**Results:**

Most participants (70%, *n* = 35) preferred to engage in shared decision making regarding cancer treatment. About half (52%, *n* = 24) did not feel that their medical team involved them in decision making and 64% (*n* = 32) felt that they were never or only sometimes listened to by the medical team. Nearly all (94%, *n* = 47) preferred to have their medical team inform them how likely treatments are to lead to cure.

**Conclusions:**

Shared decision making was the preferred mode of treatment decision making by the majority of the surveyed cancer patients in Malawi. Cancer patients in Malawi may have similar preferences to cancer patients in other low-resource settings regarding decision making and communication.

## Introduction

In resource-rich settings, communication in oncology care has become patient centered, encouraging active patient participation in their care.^1,2^ Oncology frequently involves sharing difficult news and shared decision making has become the preferred mode of decision making.^3–5^

In sub-Saharan Africa (SSA), where cancer incidence is growing, health care delivery has historically been driven by providers and patient-centered care is a relatively new concept.^6–8^ Poor patient–clinician communication is frequently identified as a contributor to delayed cancer diagnosis and incomplete treatment courses.^9–11^ Furthermore, in other resource-constrained settings, patients have indicated a preference for engaging in shared decision making regarding serious illnesses such as cancer.^7,12,13^ Communication values are driven by cultural values, underscoring the importance of assessing communication priorities for patients in different settings.^8,14–17^

There are no existing data from Malawi exploring decision-making preferences among oncology patients. Understanding such preferences can encourage providers to engage in shared decision making and improve patient-centered communication. This study was conducted to initially assess oncology patients' decision making and communication preferences.

## Methods

### Setting and participants

Kamuzu Central Hospital (KCH) in Lilongwe, Malawi, is a tertiary referral center serving the northern and central regions with a catchment of ∼9 million people. It is the national cancer center with the adult oncology clinic seeing 45 patients per week and 720 new cancer diagnoses each year. The clinic is staffed by government-employed and University of North Carolina Project-Malawi (UNCPM) providers, who conduct prospective clinical research studies.^18–26^ Participants were eligible to participate in this study if they were older than 18 years and receiving oncology care at this clinic. We recruited a convenience sample of 50 participants.

### Survey development and data collection

Basic sociodemographic and cancer diagnosis and treatment data were collected. To assess preferences and experiences of medical decision making, prognosis information preferences, and management of emotional challenges in clinical care, six questions were developed using existing literature by the research team, including clinicians experienced in engaging with this population.^27^ Each question had three response items. The survey was developed in English and translated into Chichewa, the national language. A research assistant trained in survey methodology read question items aloud to participants face to face, due to low literacy in this population.^28^

### Analysis

To assess agreement in preferences and experiences, three question response items were collapsed into binary variables for analysis. Questions regarding patient agreement were collapsed from “yes, definitely” and “yes, somewhat” into “yes” versus “no.” The question regarding patient preference for their involvement in cancer treatment decision making was collapsed from “you prefer to mainly make the decisions” and “you prefer for you and your doctor to make the decisions together” into “you prefer to be involved in decision-making” versus “you prefer your doctor to mainly make the decisions.”

Survey data were summarized using simple descriptive statistics. Differences in decision involvement preferences by demographic characteristics were assessed with Fishers Exact Test (*α* = 0.05). All analyses were conducted using R version 4.0.2 (Vienna, Austria) or SAS version 9.4.

## Results

### Sociodemographic information

Fifty participants completed the surveys. The majority were women (*n* = 37, 74%) with a median age of 43 years (interquartile range [IQR]: 36, 48). Most participants (*n* = 44, 90%) identified as Christian, and 34% (*n* = 17) of participants had any secondary school education (Table 1). The sociodemographic information of the study population reflects that of the broader cancer population served by KCH.

**Table 1.** Demographics

Question	***n*** (%)
Gender	
Male	13 (26)
Female	37 (74)
Age (years)^a^	43 (36, 48)
Home roof	
Thatch roof	24 (48)
Tin roof	26 (52)
Home flooring^b^	
Earthen floor	31 (63)
Tile or concrete floor	18 (37)
Home water source^b^	
Tap	19 (39)
Borehole	28 (57)
Well	2 (4)
Electricity inside the home^b^	
No	34 (69)
Yes	15 (31)
SES proxy^b,c^	
No housing indicators	19 (39)
At least one housing indicator	30 (61)
Religion^b^	
Christian	44 (90)
Islam	4 (8)
None	1 (2)
Education level	
Less than primary	8 (16)
Completed primary	25 (50)
Completed secondary	13 (26)
Completed more than secondary	4 (8)

^a^ Median (IQR).

^b^ Missing (*n* = 1).

c Housing indicators include having a tin roof, tile or concrete floor, tap water, and electricity inside the home.

IQR, interquartile range; SES, socioeconomic status.

### Cancer care

The median time since cancer diagnosis was 14 months (IQR: 5, 31). Most respondents had breast cancer (*n* = 18, 36%) or lymphoma (*n* = 18, 36%). Most participants (*n* = 38, 76%) knew their current number of chemotherapy cycles (median: 7; IQR: 4, 8), but the majority (*n* = 40, 80%) were unsure of the number of remaining chemotherapy cycles (Table 2). Slightly more than half (*n* = 28, 56%) had care provided by KCH providers, with the remainder (*n* = 22, 44%) receiving care from UNCPM providers.

**Table 2.** Cancer Care

Question	***n*** (%)
Months since diagnosis^a,b^	14 (5, 31)
Cancer type	
Breast cancer	18 (36)
Cervical cancer	6 (12)
Esophageal cancer	2 (4)
Non-Hodgkin's lymphoma	1 (2)
Leukemia	1 (2)
Lymphoma	18 (36)
Multicentric Castleman disease	1 (2)
Multiple myeloma	1 (2)
Skin cancer	2 (4)
Know the number of current chemo cycles	
Known	38 (76)
Unknown	12 (24)
Current chemotherapy cycle number^a,c^	7 (4, 8)
Know the number of remaining chemo cycles	
Known	10 (20)
Unknown	40 (80)
Remaining chemotherapy cycles^a,c^	3 (0, 4)
Patient's Cancer Care Provider	
University of North Carolina	22 (44)
KCH	28 (56)

^a^ Median (IQR).

^b^ Missing (*n* = 3).

^c^ Among those who knew the current or remaining cycles.

KCH, Kamuzu Central Hospital.

### Medical decision-making preferences

The majority of participants (*n* = 35, 70%) reported they preferred their doctor make decisions regarding their care together with them. Only 24% (*n* = 12) reported preferring that the medical team make decisions regarding treatment plans without their input and only 6% (*n* = 3) preferred making decisions themselves (Table 3). No differences were found in preference when compared across gender, education, and socioeconomic status (data not shown). About half of participants (48%, *n* = 22) reported feeling at least somewhat involved by the medical team in decision making related to their care (Table 3).

**Table 3.** Patient Care Preferences

Cancer care preferences	***n*** (%)
How do you prefer to make decisions about your cancer treatment?	
You prefer to mainly make the decisions	3 (6)
You prefer for you and your doctor to make the decisions together	35 (70)
You prefer your doctor to mainly make the decisions	12 (24)
Would you like for your medical team explain to you how likely your cancer treatments will lead to cure or whether your own life is at risk?	
Yes, definitely	47 (94)
Yes, somewhat	0 (0)
No	3 (6)

Cancer care experiences	***n (%)***
Did your medical team involve you in making decisions about your care?^a^	
Yes, definitely	21 (46)
Yes, somewhat	1 (2)
No	24 (52)
During your cancer treatment at KCH, did your medical team explain to you how likely your cancer treatments will lead to a cure or whether your own life is at risk in a way that was easy to understand?	
Yes, definitely	41 (82)
Yes, somewhat	0 (0)
No	9 (18)
During your cancer treatment at KCH, did your medical team talk about any emotional problems, such as anxiety or depression, related to your cancer or chemotherapy treatments?^b^	
Yes, definitely	34 (69)
Yes, somewhat	2 (4)
No	13 (27)
During your cancer treatment at KCH, how often did your medical team listen carefully to you?	
Never	26 (52)
Sometimes	6 (12)
Always	18 (36)

^a^ Missing (*n* = 4).

^b^ Missing (*n* = 1).

### Prognosis information preferences and effectiveness of delivery

Regarding prognosis, 94% (*n* = 47) of respondents reported they would strongly prefer (responded “yes, definitely”) their medical team discuss how likely treatments are to lead to cure or whether their lives were considered to be at risk, with 6% (*n* = 3) of respondents answering they would not want this information addressed. When surveyed, 82% (*n* = 41) of participants felt that their medical team had explained this to them in a way that was easy to understand. Related to information preferences and effectiveness of delivery, 18% (*n* = 9) did not feel that their medical team explained how likely their cancer treatments would lead to a cure or whether their own life was at risk in a way that was easy to understand (Table 3).

### Managing emotional challenges

Most participants felt that their medical team talked with them about emotional challenges: 69% (*n* = 34) responded “yes, definitely” and 4% (*n* = 2) responded “yes, somewhat,” whereas 27% (*n* = 13) answered that the medical team did not talk about any emotional challenges (Table 3). Less than half, 36% (*n* = 18), felt that their medical team always listened to them, 12% (*n* = 6) felt that the medical team sometimes listened to them, and 52% (*n* = 26) felt the medical team never listened to them (Table 3).

### Agreement in preferences and experiences

**FIG. 1.**
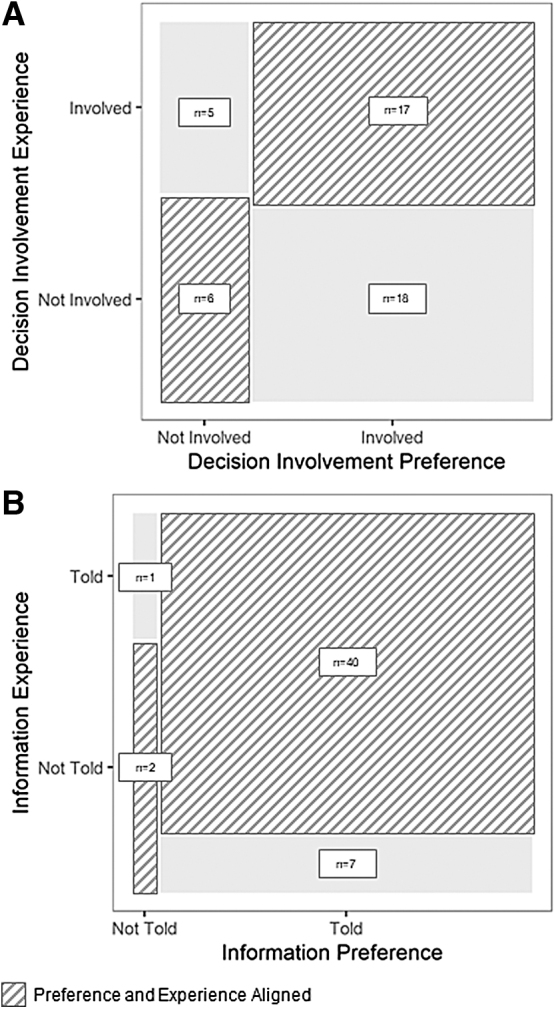
Patient preference and experience about being involved in their care and being given information about their potential outcomes. **(A)** Decision involvement experience question: How do you prefer to make decisions about your cancer treatment? Decision involvement preference question: Did your medical team involve you in making decisions about your care? **(B)** Information experience question: Would you like for your medical team explain to you how likely your cancer treatments will lead to cure or whether your own life is at risk? Information preference question: During your cancer treatment at KCH, did your medical team explain to you how likely your cancer treatments will lead to a cure or whether your own life is at risk in a way that was easy to understand? KCH, Kamuzu Central Hospital.

In total, 50% (*n* = 23) were involved in their cancer care in the way they preferred (not involved: *n* = 6, 13%; involved: *n* = 17, 37%). The other 50% (*n* = 23) either wanted to be involved in their cancer care decisions and felt they were not (*n* = 18, 39%) or did not want to be involved in their cancer care decisions and were involved (*n* = 5, 11%) (Fig. 1A). In addition, 84% (*n* = 42) received the level of information they wanted about the likelihood of cure (wanted to know and were told: *n* = 40, 80%; did not want to know: *n* = 2, 4%). The other 16% either wanted to know and were not told (*n* = 7, 14%) or did not want to know and were told (*n* = 1, 2%) (Fig. 1B).

## Discussion

In Malawi, oncology medical decision making has historically been predominately directed by medical teams. As patient communication preferences are influenced by cultural values, it is important to consult patients regarding how clinical information is shared, especially when information is complex and emotionally challenging.^5,14^ This is the first study assessing oncology decision-making preferences in Malawi.

We found that many participants prefer to engage in shared decision making, the default in high-income settings, with most opting for patient-centered communication in their clinical communication.^1^ This is also an emerging preference in obstetrics care within Malawi.^29^ Preference for shared decision making has similarly been found in other resource-constrained settings, with patients wanting more information than was historically perceived and to be involved in treatment decision making.^7,8,13^

In addition, we found that 97% of participants wanted to be told prognostic information, similar to other studies in high-resource settings and elsewhere in SSA where most patients identified that receiving this prognostic information is important to them.^7,13^ This is in contrast to perceptions of family members and health care providers that patients do not want or should not be told this information.^13^ These results highlight the importance of identifying patients' preferences when communicating prognostic information.

There were differences in preferences and experiences of the participants, with about half of the surveyed participants not involved in their cancer care decision making like they wanted, suggesting that the approach to communication and care does not always align with patients' preferences, as has been seen elsewhere in SSA.^13^ This may be partially due to the existing social context as Malawi has a patriarchal family-oriented culture, and patients have not always been included in medical decision making. Cancer diagnoses in Malawi have associated stigma and misconceptions, and families often opt not to disclose seriousness of illness.^11,26^ Our findings suggest that it remains important to ask individuals their communication and decision-making preferences.

This study is limited by the convenience sampling, as participants may have been at different places in their treatment time course, which presumably could affect preferences on communication values. However, our convenience sample is generally representative of the patient population receiving care at KCH, both in sociodemographic characteristics and in cancer diagnosis and treatment. The survey is also not validated, and it is possible that respondents did not understand some of the questions and concepts. Further research should be done on patient-centered communication preferences and should also include family preferences and health care providers' viewpoints on communication.

## Conclusions

Shared decision making is the preferred mode of treatment decision making by a majority of surveyed cancer patients in Malawi. Cancer patients in Malawi may have similar preferences to cancer patients in other low-resource settings regarding decision making and communication. This study highlights the importance of oncology providers engaging their patients and understanding their preferences on decision making and involvement in their care.

## Abbreviations Used

IQR: interquartile range
KCH: Kamuzu Central Hospital
SSA: sub-Saharan Africa
UNCPM: University of North Carolina Project-Malawi
